# Efficacy and Safety of Immune Checkpoint Inhibitor in Advanced Esophageal Squamous Cell Carcinoma: A Meta-Analysis

**DOI:** 10.3389/fonc.2021.777686

**Published:** 2021-12-21

**Authors:** Yi-Min Gu, Qi-Xin Shang, Yue Zhuo, Jian-Feng Zhou, Bo-Wei Liu, Wen-Ping Wang, Guo-Wei Che, Long-Qi Chen

**Affiliations:** ^1^ Department of Thoracic Surgery, West China Hospital of Sichuan University, Chengdu, China; ^2^ West China School of Medicine, Sichuan University, Chengdu, China

**Keywords:** esophageal squamous cell carcinoma, immune checkpoint inhibitor, anti-tumor activity, survival, adverse event

## Abstract

**Background:**

The published evidence from several randomized controlled clinical trials of immunotherapy for advanced esophageal squamous cell carcinoma has shown promising results. This study aimed to investigate the efficacy and safety of immune checkpoint inhibitor treatment in esophageal squamous cell carcinoma.

**Methods:**

PubMed, Web of Science, Cochrane Library, and Embase databases were searched for relevant articles published before December 30, 2020. The data for efficacy and safety of immune checkpoint inhibitor treatment were subjected to meta-analysis.

**Results:**

Seven clinical trials comprising 1733 patients were included. The results showed that immune checkpoint inhibitor treatment as second- or later-line treatment was associated with an increased risk of the objective response rate (relative risk: 1.82, 95% confidence interval: 0.82–4.04; P=0.002) and median overall survival (hazard ratio: 0.75, 95% confidence interval: 0.67–0.85; P<0.001) compared with chemotherapy in locally advanced or metastatic esophageal squamous cell carcinoma. Moreover, immune checkpoint inhibitor treatment was associated with significant improvement in median overall survival (hazard ratio: 0.61, 95% confidence interval: 0.48–0.77, P<0.001) compared with chemotherapy in the programmed death-ligand 1 (PD-L1)-positive population. However, immune checkpoint inhibitor treatment was also effective in all patients independent of PD-L1 expression. The most common grade ≥3 treatment-related adverse events with immune checkpoint inhibitor therapy were anemia, asthenia, rash, fatigue, decreased appetite, diarrhea, pneumonia, decreased neutrophil count, and vomiting. Patients undergoing immune checkpoint inhibitor therapy was associated with a decreased risk of treatment-related adverse events (relative risk: 0.82, 95% confidence interval: 0.62–1.08; P<0.001) and grade ≥3 treatment-related adverse events (relative risk: 0.50, 95% confidence interval: 0.42–0.60; P<0.001) compared with those undergoing chemotherapy.

**Conclusions:**

Immune checkpoint inhibitors as second- or later-line therapy may improve overall response rate and overall survival but not all oncological outcomes for patients with locally advanced or metastatic esophageal squamous cell carcinoma. Patients treated with immune checkpoint inhibitors might experience fewer treatment-related adverse events of any grade, but specifically grade ≥3, compared with those treated with chemotherapy.

## Introduction

Esophageal cancer is the seventh most common malignant tumor and the sixth leading cause of cancer death worldwide (1). To date, the optimal therapy for local advanced esophageal cancer has consisted of multidisciplinary therapy involving neoadjuvant chemotherapy or chemoradiotherapy plus surgery. Despite improvements in treatment, the long-term survival for patients with advanced esophageal cancer is still unsatisfactory (2, 3).

Immunotherapy, with agents such as immune checkpoint inhibitors (ICIs), cancer vaccines, and adoptive T-cell therapy, has recently increased hope for improved survival outcomes in patients with esophageal cancer (4–7). ICI therapy has dramatically changed the treatment of melanoma and advanced non-small cell lung cancer (8–12). In the past few years, published evidence from randomized controlled clinical trials (RCTs) has shown promise for treatment of esophageal squamous cell carcinoma (ESCC) (13–15). The two most common types of esophageal cancer are ESCC and esophageal adenocarcinoma (EAC), the incidence of which can vary by region, with the highest rate of EAC occurring in Western countries and of ESCC occurring in East Asian countries. There are clear differences between the etiology, molecular biological features, and prognosis of ESCC and EAC (16, 17). ESCC with a high level of tumor mutations appeared to be more sensitive to treatment than EAC (18). The randomized phase 3 trial KEYNOTE-181 showed that patients with ESCC treated with anti-programmed death-ligand 1 (PD-L1) antibody therapy tended to survive longer than the overall patient population but did not make a direct comparison between treatments (13). Given the high prevalence of ESCC in East Asia and the shortage of effective treatment options for advanced ESCC, conventional chemotherapy is far from satisfactory. Thus, there is an urgent need for the development of novel and effective treatments for advanced ESCC.

This study aimed to perform a meta-analysis to assess the efficacy and safety of ICI treatments for patients with advanced ESCC. Findings from this meta-analysis may be helpful in guiding ICI treatment for patients with ESCC.

## Methods

### Study Search

We conducted a systematic literature review according to the Preferred Reporting Items for Systematic Reviews and Meta-Analyses (PRISMA) 2009 guidelines (19). Two authors independently searched PubMed, Web of Science, Cochrane Library, and Embase for relevant clinical trials published before December 31, 2020. The search keyword terms were as follows: ((esophageal neoplasm [MeSH Terms]) OR ((((((esophageal squamous cell carcinoma) OR (oesophageal squamous cell carcinoma)) OR (squamous cell carcinoma of esophagus)) OR (squamous cell carcinoma of oesophagus)) OR (esophageal cancer)) OR (oesophageal cancer))) AND (immunotherapy [MeSH Terms]) OR ((((((((((((immune checkpoint inhibitor) OR (PD-1)) OR (PD-L1)) OR (Nivolumab) OR (Pembrolizumab)) OR (Camrelizumab)) OR (SHR-1210) OR (Toripalimab)) OR (Ipilimumab)) OR (Avelumab) OR (Atezolizumab)) OR (Durvalumab))).

### Study Selection, Data Extraction, and Quality Assessment

The inclusion criteria were clinical trials that included ICI monotherapy as second- or later-line treatment for patients with advanced or metastatic ESCC. Hazard ratio (HR) and relative risk (RR) for antitumor activity, survival outcomes, and safety indicators were available. Two researchers independently selected studies and extracted data; if there were any questions, another senior researcher was invited to discuss these. The following information was extracted from the selected articles: author, year, study name, study design, participant characteristics, sample size, and interventions. A quality appraisal of three randomized trials was performed using the Cochrane Risk of Bias tool (20).

### Statistical Analysis

HR, RR, and their associated 95% confidence interval (CI) were extracted from each article and combined to estimate the prognostic value. HR or RR <1 indicated a better oncologic outcome in patients with esophageal cancer treated with ICI than in those treated with chemotherapy. The Q test and I-squared statistic were used to assess the heterogeneity of the included studies. Pooled estimates of HR or RR were calculated initially using a fixed-effect model. If significant heterogeneity existed (P<0.10 or I2>50%), a random-effect model was used. Publication bias was evaluated by both Begg’s and Egger’s tests. All P-values were two-sided and significant publication bias was defined as P<0.05. Subgroup analyses were performed on the basis of which anti-PD-L1 antibody was used. All statistical analyses were performed using Stata/SE 12.0 software (StataCorp. LLC, version 12.0, College Station, TX, USA).

## Results

### Study Identification and Study Characteristics

**Figure 1:**
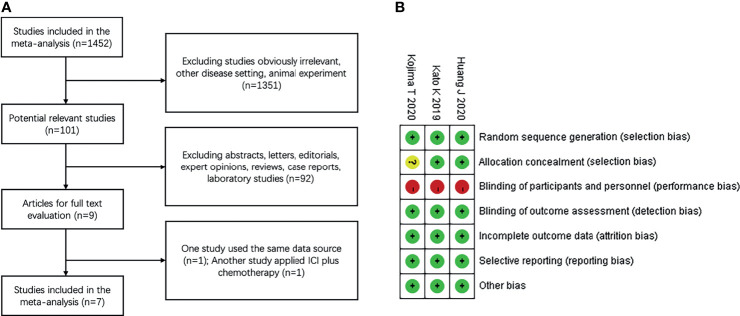
Study identification and risk of bias **(A)** Flow diagram of identification of relevant studies; **(B)** Summary of risk of bias summary of randomized controlled trials. + low risk,? unclear risk, – high risk.

Our search screened 1452 eligible studies and identified nine clinical trials; one study by Kato et al., 2020 (21) used the same dataset as that reported by Kudo et al., 2017 (22). In another trial by Zhang et al., 2020 (23) the study arm applied ICI combined with chemotherapy, which did not meet the inclusion criteria. Finally, a total of seven articles (13–15, 22, 24–26) were included in this meta-analysis. The flow diagram for identifying relevant studies is shown in **Figure 1A**.

All the included studies were published in peer-reviewed journals between 2010 and 2019 and were performed in eight countries (Japan, China, France, South Korea, USA, France, United Kingdom, and Germany). Of these clinical trials, three were multicenter, open-label, phase 3 RCTs (13–15) comparing ICI monotherapy vs. chemotherapy, and four were single-arm, prospective, phase 1–2 trials (22, 24–26) applying ICI monotherapy. Four trials enrolled patients with ESCC, three enrolled patients with both ESCC and EAC. All trials investigated anti-PD-L1 antibody therapy (three with pembrolizumab, two with camrelizumab, and two with nivolumab). A comprehensive outline of the characteristics of the included clinical trials are presented in **Table 1**. Three randomized trials reported the sample size assessment and follow-up time, but the method used for study allocation concealment in one study was unclear (**Figure 1B**). Because of a lack of appropriate evaluation tools, the risk of bias in the four single-arm trials was not estimated.

**Table 1:** Baseline characteristics of included studies.

Author	Study name	Study design	Participants characteristics	Sample size	Study arm (N)	Control arm (N)
Kojima T, (13)	KEYNOTE-181	RCT phase 3	Advanced/metastatic ESCC or EAC that progressed after one prior therapy	628 (401 ESCC & 227EAC)	Pembrolizumab	Chemotherapy with paclitaxel, docetaxel, or irinotecan (314)
					200 mg/3 weeks i.v. (314)	
Huang J, (14)	ESCORT	RCT phase 3	Advanced/metastatic ESCC; ECOG 0-1; and had progressed on, or were intolerant to, first-line standard therapy	448	Camrelizumab	Chemotherapy with docetaxel 75 mg/m2/3 weeks or irinotecan 180 mg/m2/2 weeks (220)
					200 mg/2 weeks i.v. (228)	
Kato K, (15)	ATTRACTION-3	RCT phase 3	Unresectable advanced or recurrent ESCC; ≥20 years old; ECOG 0–1; and who were refractory or intolerant to previous chemotherapy and had a life expectancy of ≥ 3 months	419	Nivolumab	Chemotherapy with paclitaxel 100 mg/m2/week or docetaxel 75 mg/m2/3 weeks (209)
					240 mg/2 weeks i.v. (210)	
Shah MA, (24)	KEYNOTE-180	Single-arm phase 2	Advanced/metastatic esophageal cancer that progressed after 2 or more lines of therapy	121 (63 ESCC & 58 EAC)	Pembrolizumab	NA
					200mg/3 weeks i.v. (121)	
Huang J, (25)	NCT02742935	Single-arm phase 1	Advanced ESCC who were refractory or intolerant to previous chemotherapy	30	SHR-1210	NA
					60 mg, with escalation to 200 mg and 400 mg/2 weeks i.v. (30)	
Doi T, (26)	KEYNOTE-028	Single-arm phase 1b	ESCC or EAC of the esophagus or gastroesophageal junction in whom standard therapy failed	23 (18 ESCC & 5 EAC)	Pembrolizumab	NA
					10 mg/kg/2 weeks i.v. (23)	
Kudo T, (22)	ATTRACTION-1	Single-arm phase 2	Advanced ESCC refractory or intolerant to fluoropyrimidine-based, platinum-based, and taxane-based chemotherapy	64	Nivolumab	NA
					3 mg/kg/2 weeks i.v. (64)	

ESCC, esophageal squamous cell carcinoma; EAC, esophageal adenocarcinoma; ECOG, Eastern Cooperative Oncology Group; NA, not available.

### Objective Response Rate (ORR) and Disease Control Rate (DCR)

The pooled ORR and DCR of ICI treatment and a subgroup analysis are summarized in **Table 2**. The pooled ORR of ICI treatment was 18.3%. The ORRs of the pembrolizumab, camrelizumab, and nivolumab ICI subgroups were 16.3%, 24.2%, and 18.5%, respectively. The pooled DCR of ICI treatment was 38.4%. The DCRs of the pembrolizumab, camrelizumab, and nivolumab subgroups were 28.0%, 46.1%, and 33.0%, respectively.

**Table 2:** ORR, DCR and OS rate in different subgroups.

Source^a^	Outcome	Heterogeneity	Rate (95% CI) %
13-15,22,24-26	ICI ORR	Fixed	18.3 (15.8-20.9)
13,24,26	Pembrolizumab ORR	Fixed	16.3 (12.3-20.2)
14,26	Camrelizumab ORR	Random	24.2 (12.4-36.0)
15,22	Nivolumab ORR	Fixed	18.5 (13.9-23.1)
14,15,22,25,26	ICI DCR	Random	38.4 (30.1-46.8)
26	Pembrolizumab DCR	——	28.0 (35.2-43.2)
14,26	Camrelizumab DCR	Fixed	46.1 (40.0-52.2)
15,22	Nivolumab DCR	Random	33.0 (23.4-42.6)
14,24,26	ICI 6-month OS rate	Random	57.1 (46.6-67.7)
24,26	Pembrolizumab 6-month OS rate	Fixed	50.8 (42.7-59.0)
14	Camrelizumab 6-month OS rate	——	63.0 (56.7-69.3)
13-15,24,26	ICI 12-month OS rate	Random	37.5 (30.6-44.4)
13,24,26	Pembrolizumab 12-month OS rate	Random	34.9 (26.0-43.7)
14	Camrelizumab 12-month OS rate	——	34.0 (27.0-40.1)
15	Nivolumab 12-month OS rate	——	47.0 (40.2-53.8)
13,24,25	PD-L1+ ORR	Random	22.2 (10.5-34.0)
13,24,25	PD-L1+ DCR	Random	48.0 (34.2-61.9)
24,25	PD-L1- ORR	Fixed	6.7 (0.9-12.4)
24,25	PD-L1- DCR	Fixed	26.9 (12.0-41.7)

a Source appertain to corresponding references; ORR, objective response rate; DCR, disease control rate; OS, overall survival; ICI, immune checkpoint inhibitor.

**Figure 2:**
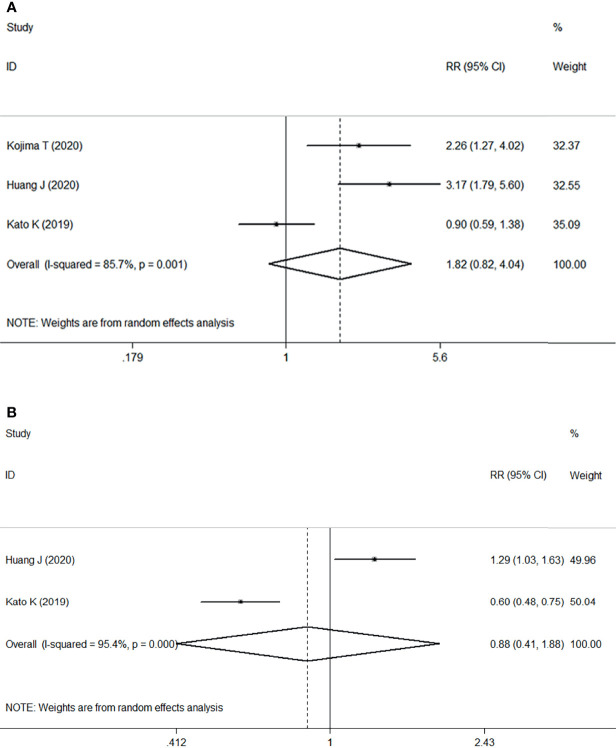
Forest plots. **(A)** Forest plots of RR comparing the objective response rate between patients treated with ICI and chemotherapy; **(B)** Forest plots of RR comparing disease control rate between patients treated with ICI and chemotherapy. RR, relative risk; CI, confidence interval; ICI, immune checkpoint inhibitor.

Three RCTs including 1268 patients demonstrated that ICI treatment was significantly associated with improvement of ORR compared with chemotherapy, with an estimated RR of 1.82 (95% CI: 0.82–4.04, P=0.002) with significant heterogeneity (I2 = 85.7%, P=0.001) (**Figure 2A**). These results suggested that ICI as second- or later-line treatment for patients with locally advanced or metastatic ESCC was associated with an increased risk of response compared with chemotherapy.

However, two studies comprising 867 patients compared the DCR between two groups, ICI versus chemotherapy. Pooled data from the two studies showed no significant difference between ICI treatment and chemotherapy, with an estimated RR of 0.88 (95% CI: 0.41–1.88, P=0.739) without apparent heterogeneity (I2 = 95.4%, P<0.001) (**Figure 2B**).

### Overall Survival Rate, Median Overall Survival (OS) and Median Progression-Free Survival (PFS)

The results of analysis of pooled 6-month and 12-month OS rate of ICI treatment and the associated subgroup analysis are also summarized in **Table 2**. The pooled 6-month OS rate of ICI treatment was 57.1%. The 6-month OS rate of the pembrolizumab and camrelizumab ICI subgroups was 50.8% and 63.0%, respectively. The pooled 12-month OS rate of ICI treatment was 37.5%. The 12-month OS rate of the pembrolizumab, camrelizumab, and nivolumab ICI subgroups was 34.9%, 34.0%, and 47.0%, respectively. The highest 6-month OS rate (63.0%) was observed in the camrelizumab subgroup and the highest 12-month OS rate (47%) in the nivolumab subgroup.

**Figure 3:**
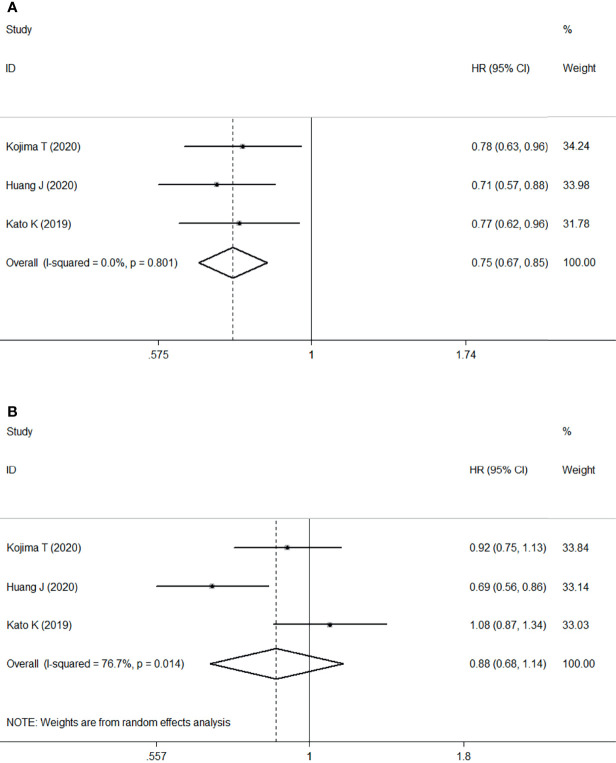
Forest plots. **(A)** Forest plots of HR comparing overall survival between patients treated with ICI and chemotherapy; **(B)** Forest plots of HR comparing progression-free survival between patients treated with ICI and chemotherapy. HR, hazard ratio; CI, confidence interval; ICI, immune checkpoint inhibitor.

Meta-analysis of three RCTs comprising 1268 patients revealed that ICI treatment improved median OS compared with chemotherapy when used as second- or later-line treatment of locally advanced or metastatic ESCC. This corresponded to a pooled HR of 0.75 (95% CI: 0.67–0.85; P<0.001) without obvious heterogeneity (I2 = 0.0%, P=0.801) (**Figure 3A**).

However, no significant difference was found in the median PFS of patients treated with ICI or chemotherapy (HR: 0.88, 95% CI: 0.68–1.14, P=0.330) with significant heterogeneity (I2 = 76.7%, P=0.014) (**Figure 3B**).

### PD-L1-Positive Tumors

**Figure 4:**
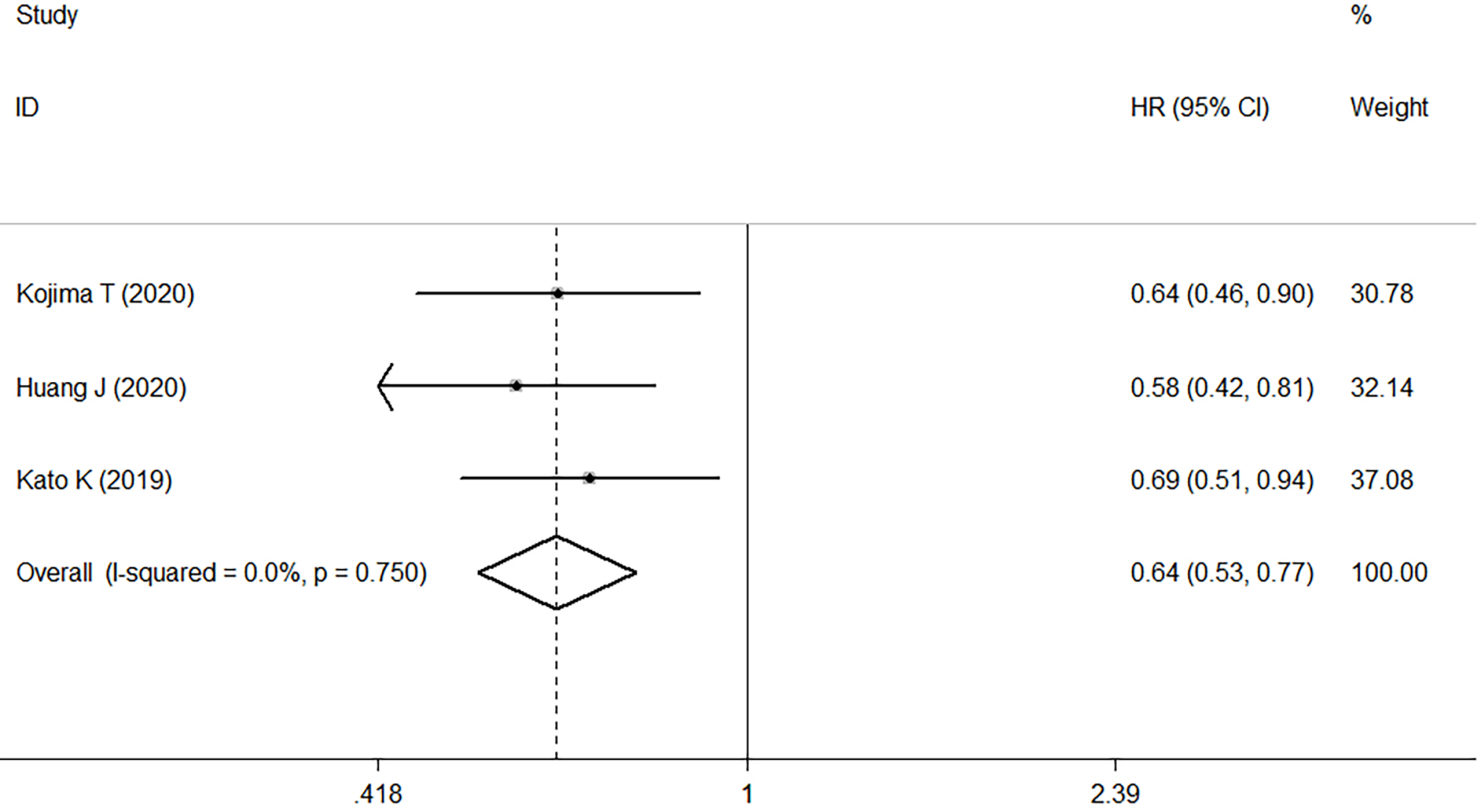
Forest plots of HR comparing overall survival between patients with PD-L1-positive tumors treated with ICI treatment and chemotherapy. HR, hazard ratio; CI, confidence interval; ICI, immune checkpoint inhibitor.

Three studies compared the antitumor activity of treatment in patients with PD-L1 positive (≥1%) and negative (<1%) tumors. The ORR and DCR of patients with PD-L1 positive tumors were 22.2% and 48.0%, while those of patients with PD-L1 negative tumors were 6.7% and 26.9% (**Table 2**). Meta-analysis of three RCTs comprising 561 patients revealed that in patients with high PD-L1 expression, median OS was improved with ICI treatment versus chemotherapy. This corresponded to a pooled HR of 0.61 (95% CI: 0.48–0.77; P<0.001) without obvious heterogeneity (I2 = 0.0%, P=0.681) (**Figure 4**).

### Treatment-Related Adverse Events (TRAEs)

We investigated the pooled incidence of any-grade TRAEs and grade ≥3 TRAEs (both total and specific events) (**Table 3**). The overall incidence of TRAEs in patients treated with ICI was 61.9%. The incidence of TRAEs in the pembrolizumab and nivolumab subgroups was 50.7% and 85.0%, respectively. However, the incidence of grade ≥3 TRAE in patients treated with ICI was 16.7%. The incidence of grade ≥3 TRAE in the pembrolizumab, nivolumab, and camrelizumab subgroups was 16.2%, 19.5%, and 15.0%, respectively. The patients in camrelizumab subgroup had the least incidence (15%).

**Table 3:** The incidence of specific TRAEs, grade ≥3 TRAEs.

TRAE Name	Subgroup	Source^a^	TRAE		Grade ≥3 TRAE	
			Heterogeneity	Rate (95% CI) %	Heterogeneity	Rate (95% CI) %
Total TRAE	Anti-PD-1	13-15,22,24-26	Random	61.9 (37.9-85.9)	Fixed	16.7 (14.4-19.0)
	Pembrolizumab	13,24,26	Random	50.7 (32.6-68.7)	Fixed	16.2 (12.9-19.6)
	Nivolumab	15,22	——	85.0 (76.3-93.7)	Fixed	19.5 (14.8-24.2)
	Camrelizumab	14,25	——	——	Fixed	15.0 (10.7-19.4)
Rash	Anti-PD-1	15,22,25,26	Fixed	10.8 (7.5-14.2)	Fixed	1.1 (-0.2-2.4)
	Pembrolizumab	26	——	13.0 (-0.7-26.7)	——	0.4 (-0.4-12.0)
	Nivolumab	15,22	Fixed	10.8 (7.1-14.5)	——	1.0 (-0.3-2.3)
	Camrelizumab	25	——	10 (-0.7-20.7)	——	0
Hypothyroidism	Anti-PD-1	13,14,24-26	Random	10.1 (5.4-14.7)	——	0
	Pembrolizumab	13,24,26	Random	7.6 (3.6-11.5)	——	0
	Camrelizumab	14,25	Fixed	16.5 (12.0-21.0)	——	0
Fatigue	Anti-PD-1	13,15,22,24-26	Fixed	9.3 (7.3-11.4)	Fixed	0.8 (0.1-1.5)
	Pembrolizumab	13,24,26	Fixed	10.6 (7.8-13.4)	——	0.6 (-0.3-1.5)
	Nivolumab	15,22	Fixed	8.0 (4.8-11.2)	Fixed	1.1 (-0.1-2.4)
	Camrelizumab	25	——	6.7 (-2.2-15.6)	——	0
Asthenia	Anti-PD-1	13,14,26	Fixed	7.0 (4.2-9.8)	Fixed	1.2 (0.3-2.1)
	Pembrolizumab	13,26	Fixed	6.7 (4.0-9.3)	——	1.3 (0.0-2.6)
	Camrelizumab	14	——	9.0 (5.3-9.6)	——	1.0 (-0.3-2.3)
Decreased appetite	Anti-PD-1	13-15,22,26	Fixed	7.0 (5.2-8.7)	Fixed	0.8 (0.1-1.5)
	Pembrolizumab	13,26	Fixed	8.0 (5.1-10.9)	——	0.6 (-0.3-1.5)
	Nivolumab	15,22	Fixed	8.2 (5.0-11.5)	Fixed	1.2 (-0.1-2.5)
	Camrelizumab	14	——	5.0 (2.2-7.8)	——	0
Diarrhea	Anti-PD-1	13-15,22,24-25	Fixed	6.0 (4.5-7.6)	Fixed	0.8 (0.2-1.4)
	Pembrolizumab	13,24	Fixed	5.3 (3.2-7.3)	Fixed	0.6 (-0.1-1.4)
	Nivolumab	15	——	11.0 (6.8-15.2)	——	1.0 (-0.3-2.3)
	Camrelizumab	14,25	Fixed	5.3 (2.6-8.1)	——	1.0 (-0.3-2.3)
Anemia	Anti-PD-1	13-15,24-25	Random	4.7 (1.4-8.1)	Fixed	1.8 (0.8-2.7)
	Pembrolizumab	13	——	2.5 (0.8-4.2)	——	1.3 (0.0-2.6)
	Nivolumab	15	——	3.0 (0.7-5.3)	——	2.0 (0.1-3.9)
	Camrelizumab	14,25	Random	7.6 (0.1-15.1)	——	3.0 (0.8-5.2)
Nausea	Anti-PD-1	13-15,25	Random	3.0 (1.8-4.1)	——	0
	Pembrolizumab	13	——	7.0 (4.2-9.8)	——	0
	Nivolumab	15	——	2.0 (0.1-3.9)	——	0
	Camrelizumab	15,22	Fixed	2.2 (0.4-4.0)	——	0
Pneumonia	Anti-PD-1	14,22,24-26	Random	2.7 (-0.5-5.9)	Random	0.5 (-0.2-1.2)
	Pembrolizumab	24,26	Fixed	6.5 (2.5-10.5)	——	2.4 (-0.3-5.1)
	Nivolumab	22	——	2.0 (-1.4-5.4)	——	0
	Camrelizumab	14,25	——	0.3 (-0.4-1.0)	Fixed	0.3 (-0.4-1.0)
Vomiting	Anti-PD-1	13,14	Random	2.0 (-0.2-4.1)	——	0.3 (-0.3-0.9)
	Pembrolizumab	13	——	3.2 (1.3-5.1)	——	0.3 (-0.3-0.9)
	Camrelizumab	14	——	1.0 (-0.2-4.1)	——	0
Neutrophil count decreased	Anti-PD-1	13-15,22	Random	1.1 (0.4-1.9)	Fixed	0.5 (-0.1-1.0)
	Pembrolizumab	13	——	0.6 (-0.3-1.5)	——	0.3 (-0.3-0.9)
	Nivolumab	15,22	——	2.0 (0.1-3.9)	Fixed	1.1 (-0.1-2.4)
	Camrelizumab	14	——	4.0 (1.5-6.5)	——	0
Alopecia	Anti-PD-1	13-15,24,26	Fixed	0.7 (-0.0-1.4)	——	0
	Pembrolizumab	13,24,26	——	0.6 (-0.3-1.5)	——	0
	Nivolumab	15	——	1.0 (-0.3-2.3)	——	0
	Camrelizumab	14	——	0	——	0

TRAE, treatment-related adverse event; ^a^Source appertain to corresponding references.

The most common TRAEs with ICI therapy of locally advanced or metastatic ESCC were rash (10.8%), hypothyroidism (10.1%), fatigue (9.3%), asthenia (7.0%), decreased appetite (7.0%), diarrhea (6.0%), anemia (4.7%), nausea (3.0%), pneumonia (2.7%), vomiting (2.0%), decreased neutrophil count (1.1%), and alopecia (0.7%). The most common grade ≥3 TRAEs with ICI therapy were anemia (1.8%), asthenia (1.2%), rash (1.1%), fatigue (0.8%), decreased appetite (0.8%), diarrhea (0.8%), pneumonia (0.5%), decreased neutrophil count (0.5%), and vomiting (0.3%).

**Figure 5:**
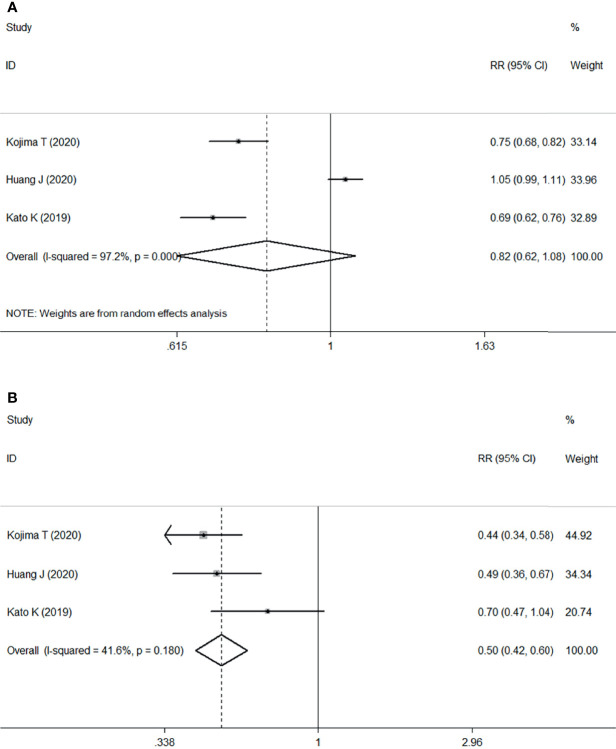
Forest plots. **(A)** Forest plots of RR comparing TRAEs between patients treated with ICI and chemotherapy; **(B)** Forest plots of RR comparing grade ≥3 TRAEs between patients treated with ICI and chemotherapy. RR, relative risk; CI, confidence interval; ICI, immune checkpoint inhibitor; TRAEs, treatment-related adverse events.

The meta-analysis of the three RCTs indicated that patients undergoing ICI therapy was associated with a decreased risk of overall TRAEs (RR: 0.82, 95% CI 0.62–1.08; *P*<0.001) (**Figure 5A**) and grade ≥3 TRAEs (RR=0.50, 95% CI 0.42–0.60; *P*<0.001) (**Figure 5B**) compared with those undergoing chemotherapy.

### Publication Bias

Publication bias was evaluated by both Begg’s and Egger’s tests (**Table 4**). All outcomes had P>0.05, except for the Egger’s test of ICI vs. chemotherapy TRAEs (P<0.05). Overall, no obvious publication bias was observed.

**Table 4:** Publication bias of different outcomes.

Outcomes	Included study numbers	Effect size	*P*	
			Begg	Egger
ICI ORR	3	logRR	1.000	0.171
ICI DCR	2	logRR	1.000	——
ICI OS	3	lnHR	1.000	0.815
ICI PFS	3	lnHR	1.000	0.967
ICI TRAEs	3	logRR	0.296	0.000
ICI grade≥3 TRAEs	3	logRR	0.296	0.077
PD-L1+ ICI OS	3	lnHR	1.000	0.505

ICI, immune checkpoint inhibitor; ORR, objective response rate; DCR, disease control rate; OS, overall survival; PFS, progression-free survival; TRAEs, treatment-related adverse events; PD-L1+, PD-L1 positive; RR, relative risk; HR, hazard ratio.

## Discussion and Conclusions

To our knowledge, this is the first meta-analysis to evaluate the efficacy and safety of ICIs (anti-PD-L1 antibody) as second- or later-line treatment for unresectable locally advanced or recurrent/metastatic ESCC. This study included seven published clinical trials including three RCTs and four single-arm trials published before December 31, 2020. The main outcomes showed that ICI therapy used as second- or later-line treatment for advanced or metastatic ESCC could increase ORR, improve OS, and decrease the incidence of any-grade TRAEs and grade ≥3 TRAEs compared with chemotherapy.

Several RCTs of ICIs have reported the clinical outcomes in patients with ESCC. The randomized phase 3 trial KEYNOTE-181 (13) reported that patients with ESCC treated with anti-PD-L1 antibody therapy showed a trend toward longer survival compared with the overall population of patients with ESCC but did not make a direct comparison. The randomized trial ATTRACTION-3 reported by Kato et al. (15) also demonstrated that median OS was significantly improved in patients treated with nivolumab compared with those treated with chemotherapy (10.9 months vs 8.4 months; HR: 0.77, 95% CI: 0.62–0.96; P=0.019). However, the effects of ICI on PFS and its antitumor activity differ between studies. The ESCORT randomized phase 3 study (14) showed that camrelizumab improved OS compared with chemotherapy as second-line therapy in Chinese patients with advanced ESCC.

Patients with PD-L1-positive tumors may derive more survival benefit with ICI therapy than with chemotherapy. However, ICI therapy was also reported to be effective in all patients independent of PD-L1 expression (14). The RCT reported by Kato et al. also observed no significant interaction between the effectiveness of ICI therapy and PD-L1 status (15). This suggests that PD-L1 might not be sufficiently specific to serve as the optimal biomarker for ICI treatment of ESCC. In advanced gastric or gastroesophageal junction cancer, high microsatellite instability and tumor mutational burden has been shown to be associated with the ORR of patients (27, 28). Studies have found that the tumor mutation burden is usually higher in ESCC than in EAC (18, 29). Further investigation of candidate biomarkers for ICI treatment is warranted.

The three randomized trials included in this meta-analysis all involved monotherapy with ICI vs. chemotherapy as a second- or later-line treatment. There are a number of unpublished clinical trials with an accessible conference abstract that evaluated the efficacy of ICI as first-line therapy and adjuvant therapy, such as the KEYNOTE-590 randomized phase 3 study (30); this showed that the median OS of patients with ESCC was longer with first-line treatment with pembrolizumab plus chemotherapy than with chemotherapy alone (12.6 months vs. 9.8 months; HR: 0.72, 95% CI: 0.60–0.88, P=0.0006). In the CheckMate 577 randomized phase 3 study (31), median DFS in patients treated with nivolumab after surgery was twice that in the placebo population (22.4 months vs. 11.0 months; HR: 0.69; 96.4% CI: 0.56–0.86; P=0.0003). The optimal timing, dosing, and combination of ICI regimens for treatment of esophageal cancer require further study.

The safety profile of ICIs showed a lower incidence of any-grade TRAEs and grade ≥3 TRAEs compared with chemotherapy. In this meta-analysis, 61.9% patients receiving ICI treatment reported TRAEs, but the probability of developing grade ≥3 TRAEs was 16.7%. Notably, the incidence of reactive cutaneous capillary endothelial proliferation after receiving camrelizumab was high (14, 23). However, no similar adverse event was noted in patients receiving pembrolizumab or nivolumab. Moreover, the incidence of treatment leading to death was almost zero. Accordingly, ICI treatment can be considered a relatively safe option.

This meta-analysis has some limitations that should be acknowledged. First, it included only seven studies comprising three RCTs and four single-arm trials. Even though no obvious publication bias was detected in the included RCTs by Begg’s or Egger’s test, the single-arm studies without a control group might introduce a potential bias. The low number of trials may limit the accuracy of the test. However, the number of 1733 included patients is relatively high, indicating reliability. Second, three studies included both patients with ESCC and those with EAC. Specific information about squamous cell carcinoma patients was provided by Kojima et al. (13), but was not available for two single-arm studies (24, 26) that included 81 ESCC patients and 63 EAC patients. Unfortunately, we do not have access to their raw data. These confounding factors may limit the interpretation of our results.

In conclusion, ICI treatment in patients with locally advanced or metastatic ESCC may improve the ORR and median OS but not all oncological outcomes, and result in a lower incidence of TRAEs compared with chemotherapy. Although ICI treatment was more effective in patients with PD-L1-positive tumors, it was also effective in all patients with ESCC independent of their PD-L1 expression. Further investigation of the optimal timing, dosing, combination of drug regimens, and candidate biomarkers for ICI treatment of esophageal cancer is warranted.

## Data Availability Statement

The original contributions presented in the study are included in the article/supplementary material. Further inquiries can be directed to the corresponding author.

## Author Contributions

L-QC and G-WC conceptualized the study, revised the manuscript and supervised the study. Y-MG and Q-XS conceptualized the study, drafted the manuscript and made the figures. Y-MG, YZ, J-FZ, B-WL and W-PW collected the literature and revised the manuscript. All authors contributed to the article and approved the submitted version.

## Conflict of Interest

The authors declare that the research was conducted in the absence of any commercial or financial relationships that could be construed as a potential conflict of interest.

## Publisher’s Note

All claims expressed in this article are solely those of the authors and do not necessarily represent those of their affiliated organizations, or those of the publisher, the editors and the reviewers. Any product that may be evaluated in this article, or claim that may be made by its manufacturer, is not guaranteed or endorsed by the publisher.
